# Toward multicomponent mesoporous single-crystalline metal oxides

**DOI:** 10.1093/nsr/nwae104

**Published:** 2024-04-05

**Authors:** Minkyeong Ban, Jinwoo Lee

**Affiliations:** Department of Chemical and Biomolecular Engineering, Korea Advanced Institute of Science and Technology (KAIST), South Korea; Department of Chemical and Biomolecular Engineering, Korea Advanced Institute of Science and Technology (KAIST), South Korea

Mesoporous metal oxide materials have attracted significant interest due to their porous frameworks and large surface areas, finding applications in sensors, catalysis and energy storage [1]. The performance of these materials is influenced by both their porous structures and the crystal phase of the frameworks [2]. In lithium-ion storage, the utilization of bulk single-crystal electrode materials enhances ionic conductivity but may trade off diffusion distance, impacting rate capability and cycle stability. Designing a mesoporous single-crystal microparticle is crucial for achieving high-performance lithium storage by combining microstructure and nanostructure advantages [3].

The two primary synthesis routes, hard-templating and soft-templating approaches, offer distinct advantages, with the latter being more straightforward, controllable and suitable for mass production [4]. Nevertheless, challenges persist in extending the soft-template approach to the synthesis of metal oxide single crystals comprising more than three components [5]. The challenge arises due to several factors, including rapid hydrolysis and condensation rate leading to macroscopic phase separation, the decomposition of surfactants at low temperatures inducing structural collapse, and the high temperature which is often needed for the crystallization of multi-metal oxides.

**Figure 1.**
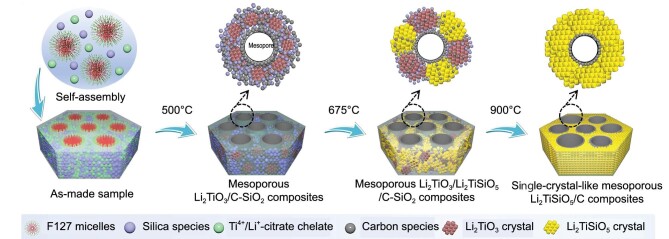
Fabrication process of single-crystal-like mesoporous Li_2_TiSiO_5_, utilizing a micelle-directed self-assembly strategy.

In a recent study led by Prof. Li, mesoporous Li_2_TiSiO_5_ exhibiting a single-crystal-like structure was fabricated using soft micelles as templates [6]. Rather than merely combining commercial precursors, Li *et al.* synthesized stoichiometric citrate Ti^4+^/Li^+^ chelate precursors. The coordination of Ti^4+^ and Li^+^ ions by carboxyl groups in citric acid not only prevented uncontrollable hydrolysis but also facilitated stable atomic dispersion. Subsequently, the conversion of citrate and silicates into rigid carbon and SiO_2_ frameworks effectively prevented structural collapse. Also, an in-depth investigation was conducted into the structural evolution of mesoporous Li_2_TiSiO_5_ exhibiting a single-crystal-like composition, correlating with escalating pyrolytic temperatures. As the temperature rose, the framework underwent a fascinating crystallization sequence, progressing from the Li_2_TiO_3_ phase, through a mixed phase of polycrystalline Li_2_TiO_3_ and Li_2_TiSiO_5_ phase, to single-crystalline Li_2_TiSiO_5_ phase (Fig. 1). The gradual decrease of pore size and volume provided evidence of the fabrication of a highly crystalline metal oxide.

Then, to explore the impact of metal oxide shape and crystallinity on lithium storage, single-crystal-like mesoporous Li_2_TiSiO_5_ was employed as the anode material for lithium-ion batteries. This material exhibited outstanding electrochemical performance, surpassing its counterparts, including bulk Li_2_TiSiO_5_ and polycrystalline Li_2_TiSiO_5_. Advantages included high-capacity retention, superior rate capability and long-term cycling performance. This result was attributed to the nanosized crystal frameworks and short Li^+^ ion diffusion lengths inherent in the single-crystal-like mesoporous Li_2_TiSiO_5_.

This leading study provides a novel method for fabricating highly crystalline mesoporous multicomponent metal oxides. This pioneering study introduces an innovative approach that has established a new standard in the fabrication of highly crystalline mesoporous multicomponent metal oxides. With its superior performance and potential for widespread applications, this discovery represents a significant leap forward in the quest for efficient energy storage solutions.
